# Supplementary material for: Mechanism underlying starvation-dependent modulation of olfactory behavior in Drosophila larva
Source: Sci Rep. 2020 Feb 20;10:3119. doi: 10.1038/s41598-020-60098-z (PMC7033209; doi:10.1038/s41598-020-60098-z)
Supplement: Supplementary file 1 — Supplementary information. [file 41598_2020_60098_MOESM1_ESM.pdf]

SUPPLEMENTARY MATERIAL

TITLE:

Mechanism underlying starvation-dependent modulation of olfactory behavior in *Drosophila* larva

AUTHORS/AFFILIATIONS:

Eryn Slankster[1], Sai Kollala[1], Dominique Baria[1], Brianna Dailey-Krempel[1], Roshni Jain[1,2], Seth R. Odell[1,3], and Dennis Mathew[1,2,3 *]

[1]Department of Biology, University of Nevada, Reno, NV, 89557; USA
[2]Cell and Molecular Biology Graduate Program, University of Nevada, Reno, NV, 89557; USA
[3]Integrated Neuroscience Graduate Program, University of Nevada, Reno, NV, 89557; USA

CONTACT INFORMATION:

*Correspondance should be addressed to:
Dr. Dennis Mathew
Tel: (775) 784 6052
Email: dennismathew@unr.edu
Address: 1664 N. Virginia St., MS: 0314, University of Nevada, Reno, NV 89557; USA

FIGURE & TABLE LEGENDS

Supplementary Figure S1. Starvation impacts expression levels of Orco but not other odor receptor genes. Normalized gene expression of *Orco* and nine *Or* genes are measured following mRNA isolation from non-starved (white bars) or starved (grey bars) larval heads. Mean ± SEM. $^{**}p = 0.0014$

Supplementary Table S1. **Primers used in RT-qPCR analysis**. Forward and reverse primer sequences used in RT-qPCR analyses, their source, and calculated primer efficiencies are shown. Expected sizes of RT-PCR products were estimated using PrimerBLAST (https://www.ncbi.nlm.nih.gov/tools/primer-blast/).

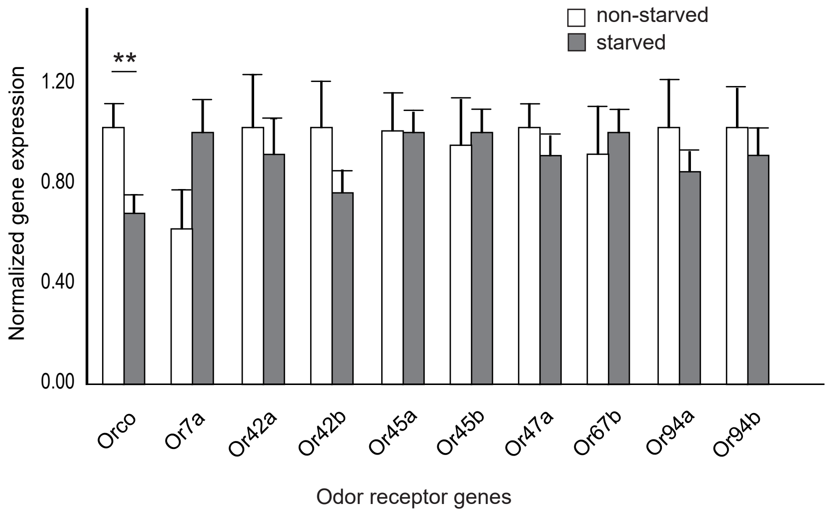

Supplementary Figure S1. Starvation impacts expression levels of Orco but not other odor receptor genes. Normalized gene expression of *Orco* and nine *Or* genes are measured following mRNA isolation from non-starved (white bars) or starved (grey bars) larval heads. Mean ± SEM. **p = 0.0014.

Table 1: Primers used in RT-qPCR analysis

Target	Forward primer sequence	Reverse primer sequence	Size (bp)	Spans exon?	Primer source	Standard Curve/Quantification Calibration				
						Primer efficiency	R^2	Dilution series	amplifyable range SD < 0.3	calibration sample used
Act42a	ATGGTAGGAATGGGACAAAAGGA	CTCAGTAAGCAAGACGGGGTG	192	No	FlyPrimerBank: PP20128	95.3%	0.997	4-fold	10ng-0.032ng	whole larvae
APPL	AGTGGAGTTCGTCTGCTGTC	TGGCGCTATTGATCTGAGCTG	101	Yes	FlyPrimerBank: PP32134	98.3%	0.996	5-fold	4ng-0.032ng	whole larvae
eGFP	GAGGGATACGTGCAGGAGAG	GATCCTGTTGACGAGGGTGT	102	No	Ma & Weake, 2013	108.9%	0.995	5-fold	10ng-0.08ng	OSNs
EF-1	GCGTGGGTTTGTGATCAGTT	GATCTTCTCCTTGCCCATCC	125	Yes	Ponton 2010	92.1%	0.996	4-fold	5ng-0.032ng	larvae heads
$GABA_BR1$	GATGTCAACAAGCAGCCAAATC	CGGGCTCACACTCACTGTC	76	Yes	FlyPrimerBank: PP15543	104.2%	0.997	5-fold	4ng-0.16ng	whole larve
$GABA_BR2$	CGCCTTGGGTCACGTTAATGA	GCATTGCACTGAGTGTCGTTC	84	Yes	FlyPrimerBank: PP22487	103.6%	0.98	5-fold	4ng-0.16ng	whole larvae
$GABA_BR3$	TGCTGCTCGGACTCTTTGAG	AGCTCCCAATTCGCTCAGAC	71	No	PrimerBLAST	94.5%	0.972	5-fold	4ng-0.16ng	OSNs
Gαl	GGTTGTGCCGTGAGTACAG	GCAGCAGTTTCACCTCCGA	112	No	FlyPrimerBank: PP5005	102.9%	0.996	5-fold	4ng-0.032ng	whole larve
GαO	GATGAAAATCATTCACGAGAGCG	CGTCGAACACCATCTTGGCAT	173	Yes	FlyPrimerBank: PP8752	98.0%	0.997	5-fold	4ng-0.032ng	whole larve
InR	AAGCGTGGGAAAATTAAGATGGA	GGCTGTCAACTGCTTCTACTG	148	No	FlyPrimerBank: PP12687	95.5%	0.998	5-fold	10ng-0.4ng	OSNs
Nervana 2	TCGAATGACTTGCCCGCGAA	GCCCTCGCACGATACCCAAA	108	Yes	Ling 2011	99.4%	0.999	4-fold	5ng-0.078ng	larvae heads
Or94b	GTACATCTGGGGCAGCCTTT	GGGTCATGGCCACCACATTA	158	No	PrimerBLAST	110.9%	0.977	3-fold	6.67ng-0.741ng	whole larvae
Orco	GATGAGGAAGCTGTTCTTTCTGG	ACCACCATTTTTACGCTGTCG	99	No	FlyPrimerBank: PP37330	106.3	0.97	5-fold	10ng-0.08ng	OSNs
Rutabaga	ACCTGCCCACATTGTGCTAC	ATGGCGTAAGCGAGGAAGAC	157	No	FlyPrimerBank: PP11257	91.2%	0.992	5-fold	20ng-0.16ng	whole larvae
sNPF-R	CCAACTGGAGCCTAACGTCG	AACTGGTTGTGAATGATCCCG	100	No	FlyPrimerBank: PP1130	86.8%	0.988	5-fold	10ng-0.08ng	larvae heads
Syt1	TCCCTATGTCAAGGTGTACTTGC	GTTGAAGACCGGACTCAGTGT	88	No	FlyPrimerBank: PP5891	104.8%	0.997	4-fold	5ng-0.0781ng	larvae heads
dILP1	CCCCGGAAACCACAAACTCT	TAAAGCCATGGGGACACACC	71	No	FlyPrimerBank: PD46040	NA		5-fold	>10ng	larvae heads
dILP2	CGAGGTGCTGAGTATGGTGTG	CCCCAAGATAGCTCCCAGGA	185	Yes	FlyPrimerBank: PP25243	90.1	0.0994	5-fold	10ng-0.08ng	larvae heads
dILP3	GTGTATGGCTTCAACGCAATG	CAGCAGGGAACGGTCTTCG	87	Yes	FlyPrimerBank: PP18236	96.3	0.991	5-fold	10ng-2ng	larvae heads
dILP4	TGGATTTACACGCCGTGTCA	GGTCTCGCACTCTAGCATCC	59	Yes	FlyPrimerBank: PD80009	NA		5-fold	>10	larvae heads
dILP5	TGCCTGTCCCAATGGATTCAA	GCCAAGTGGTCCTCATAATCG	78	Yes	FlyPrimerBank: PP18945	100.5	0.997	5-fold	10ng-0.08ng	larvae heads
dILP6	CCCTTGGCGATGTATTTCCCA	CTTGCAGCACAAATCGGTTAC	80	Yes	FlyPrimerBank: PP27008	70.1	0.997	5-fold	10ng-0.4ng	larvae heads
dILP7	CCTGGCTGCACGTGAACTAT	TGGATGGACAATACTCGGCG	135	No	FlyPrimerBank: PD46038	NA	0.973	5-fold	10ng-2ng	larvae heads
dILP8	CGACAGAAGGTCCATCGAGTT	GTGATGCTTGTTGTGCGTTTT	76	Yes	FlyPrimerBank: PP21172	96.6	0.986	5-fold	10ng-0.4ng	larvae heads
GAD1	TGAATCCCAACGGGTATAAACTG	TCACTGTTGTGGGCATGAGAT	75	Yes	FlyPrimerBank: PP383	95.2	0.999	5-fold	10ng-0.08ng	OSNs

Supplementary Table S1. Primers used in RT-qPCR analysis. Forward and reverse primer sequences used in RT-qPCR analyses, their source, and calculated primer efficiencies are shown. Expected sizes of RT-PCR products were estimated using PrimerBLAST (https://www.ncbi.nlm.nih.gov/tools/primer-blast/).
